# Gut Microbiome Implication and Modulation in the Management of Recurrent Urinary Tract Infection

**DOI:** 10.3390/pathogens13121028

**Published:** 2024-11-21

**Authors:** Mattia Brigida, Angela Saviano, Carmine Petruzziello, Luca Luigi Manetti, Alessio Migneco, Veronica Ojetti

**Affiliations:** 1 Gastroenterology Department, Policlinico Tor Vergata, 00133 Rome, Italy; 2 Emergency Department, Ospedale Policlinico A. Gemelli, 00168 Rome, Italy; 3 Emergency Department, Ospedale San Carlo di Nancy, GVM Care & Research, 00165 Rome, Italy; 4 Internal Medicine Department, San Carlo di Nancy Hospital, GVM Care & Research, 00165 Rome, Italy; 5 Department of Internal Medicine, UniCamillus International University of Health Sciences, 00131 Rome, Italy

**Keywords:** urinary tract infection, gut microbiota, probiotics, *Escherichia coli*, antibiotics, urobiome, microbiome, UTI, gut–bladder axis

## Abstract

Urinary tract infections (UTIs) are one of the most common bacterial infections, affecting more than 150 million people each year in the world. UTIs have grown exponentially in the last few years. They represent a major load for both individuals and society. The highest incidence (about 55–60%) concerns women. Many pathogens are involved in UTIs, most of which are derived from the gut. Recent studies, together with recent diagnostic techniques (such as quantitative culture of urine or next-generation sequencing), have improved the knowledge of microbial communities in the urinary tract. It turned out that gut dysbiosis is strictly involved in the pathogenesis of UTIs. In particular, the human gut is the natural habitat for *Escherichia coli* (*E. coli*), the main bacterium responsible for UTIs. The overgrowth of *E. coli* pathogenic strains represents a risk factor for them. Furthermore, the human gut microbiota acts as a “global reservoir” for genes conferring resistance to clinically relevant antibiotics, thus influencing the treatment of UTIs. In addition, differently from the past, the idea of a sterile urinary environment has been replaced by the characterization of a urinary microbiome. The aim of our review is to explore recent studies on the association between gut microbiota and urinary microbiome and to summarize the current knowledge about the effects of interactions between gut and urinary microbial communities in the pathogenesis of UTIs, considering UTIs more as a “gut disease” and not only a urinary disease and providing new insight into the therapeutic options such as the use of probiotics.

## 1. Introduction

Urinary tract infections (UTIs) are common bacterial infections worldwide that range from uncomplicated cystitis to severe urosepsis. It is estimated that UTIs mainly affect females (3.5 times more than males), with a peak at about 35–40 years old [1]. From 65 years old, the complications and mortality are almost comparable between the two sexes. UTIs represent a significant burden for both individuals and healthcare systems [2]. UTIs are usually caused by the uropathogenic *Escherichia coli* (*E. coli*—UPEC), which is resident in the human gut and can move and reach the urinary tract causing infection. This process is facilitated by host behaviors (such as sexual intercourse, physical manipulation), genetics, and pathogens. Other bacteria responsible for UTIs are *Enterobacter*, *Klebsiella*, *Staphylococcus*, *Proteus*, and *Enterococcus* [3,4]. People can experience recurrent UTIs, defined as two episodes of acute symptomatic bacterial cystitis within the last 6 months, or three episodes within the previous year. Symptoms include dysuria, frequency, urgency, or suprapubic pain associated with more than 100,000 colony-forming units (CFU/mL). Recurrent UTIs are more common in women compared to men, and they can range from uncomplicated forms to complicated ones. It is estimated that about one-third of women have an uncomplicated UTI before 24 years of age [5,6]. Then, the prevalence of at least one symptomatic UTI in women becomes more than 50%, and about 25% of women have a recurrence during 6 months of follow-up after antibiotic treatment of the initial UTI. After 55 years old, more than 50% of women experience a recurrence of UTIs within one year [3]. Patients with a history of complicated UTIs are at high risk of developing recurrent forms, especially those suffering from multiple comorbidities (such as diabetes) or patients who have a permanent catheter or previous septic shock. Moreover, therapeutic urological interventions can also have a role in increasing the epidemiological data [7]. The treatment, mainly based on antibiotics, can lead to resistant strains, requiring prolonged therapies and incurring high costs for the healthcare system. Studies in the literature underline that patients suffering from UTIs typically have high concentrations of UPEC in their gut, and this is considered a cause of recurrence [8,9]. *E. coli* normally lives in the human gut, but it can reach the urinary tract through migration or external contamination. A study by Magruder et al. observed that an increased abundance of *E. coli* in the gut was positively associated with an increased frequency of recurrent UTIs [3]. There is evidence stating that that the human gut microbiota was implicated in *Uropathogenic Escherichia coli* (UPEC) urinary tract colonization during episodes of recurrent UTIs [10,11]. It was assumed that the gut turned out to be a reservoir for UPEC. The latter can asymptomatically colonize both the gut and, in case of intestinal barrier dysfunction, the urinary tract, predisposing patients to recurrent UTIs [12]. Although the relationship between the gut microbiota and UTIs in adults and children continues to be studied, recent data revealed the presence of distinct dynamic microbial communities in the urinary system, known as the urinary microbiome [13]. Differently from the past, the idea of a sterile urinary environment has now been overcome. These other urinary microbial communities can be influenced by factors such as lifestyle, age, sexuality, poor hygiene, metabolic changes (e.g., metabolic diseases or conditions such as menopause), exposure to environmental metals or antibiotics, poor diet, and protein impairment that depletes microbial diversity [14,15]. The urobiome (i.e., urinary microbiome) is less diverse than its gut counterpart. It is usually dominated by a single genus or species [16,17,18]. On the contrary, the gut microbiota has much diversity, with hundreds of different species of microorganisms. This diversity is important for overall health, as disruptions to the gut balance have been linked to a variety of inflammatory diseases, such as UTIs and/or recurrent UTIs. Microbial dysbiosis, as said before, predisposes individuals to the invasion of uropathogenic bacteria.

In this review, our aims are to analyze studies about the association between the gut microbiota and the urinary microbiome and the effects of their interactions and lack of balance and finally to discuss therapeutic interventions (i.e., how the modulation of the microbiota has been considered as a future intervention strategy for recurrent UTIs) [19]. In this context, the field of probiotics has been under investigation to identify the most specific and effective strains to restore and protect the health of the human urothelium.

We performed a narrative review, including articles in the English language published in the last twenty years. We searched terms such as “urinary tract infection” OR “UTIs” OR “recurrent urinary tract infection” OR “acute cystitis” OR “acute pyelonephritis” AND “gut microbiota” OR “urinary microbiota”, OR “microbiome”, OR “leaky gut” OR “gut bladder-axis” AND “probiotics”. Throughout a quality assessment, we included relevant original research, reviews, clinical trials, abstracts published in journals provided with an impact factor and available on PubMed^®^, Up-To-Date^®^, or Web of Science^®^. Our exclusion criteria were lack of pertinence, article published > 20 years ago, or article published in a journal without an impact factor. Therefore, out of 192 articles initially found using the aforementioned keywords, we excluded 28 articles due to lack of pertinence (e.g., microbiota and urinary tract cancer), 17 articles published > 20 years ago, and 26 articles that were published in journals without an impact factor. Hence, the final number of included articles is 120, of which the most relevant are resumed in Table 1. No ethical approval was required.

### 1.1. Leaky Gut and Bacterial Translocation to the Bladder

The gastrointestinal (GI) tract plays a critical role in preventing the translocation of bacteria and other harmful substances into the systemic circulation. “Leaky gut” refers to the condition where the intestinal barrier becomes compromised, leading to increased permeability. This phenomenon has been associated with various systemic diseases, including urinary tract infections (UTIs), as bacterial translocation to the bladder is increasingly recognized as a potential contributing factor [20]. This chapter delves into the mechanisms underlying leaky gut and its implications for bacterial translocation to the bladder, highlighting the clinical relevance of these findings. The intestinal barrier, composed of tightly joined epithelial cells, regulates the passage of substances and prevents the entry of pathogens [21]. Factors such as chronic inflammation, dysbiosis, and dietary influences can disrupt the integrity of these tight junctions, leading to leaky gut syndrome. When the gut barrier is compromised, bacteria, endotoxins, and antigens can cross into the bloodstream, potentially causing systemic inflammation and increasing the risk of UTIs [22]. In particular, the condition of dysbiosis determines the overgrowth of bacteria responsible for urinary tract colonization [23]. Techniques such as 16S rRNA rapid next-generation gene sequencing (NGS) and expanded quantitative urine culture (EQUC) have led to the identification of more than 50 genera and 100 species in the urinary tract [24]. Furthermore, some pathogenic strains of *E. coli* encode genes that lead to the production of adhesins, toxins, flagella, etc., further contributing to pathogenicity. A study by Choi et al. analyzed 125 patients with UTIs, collecting stool samples and analyzing antimicrobial resistance genes, taxonomic composition, and phenotypic resistance [10]. The gut microbiome of patients with UTIs was compared with the gut microbiome of healthy individuals. The authors found that the risk of recurrent UTIs was not independently associated with clinical presentation. The gut microbiota of patients with a diagnosis of UTI was distinct from healthy individuals in both taxonomic composition and antimicrobial resistance genes. The authors identified 11 different taxa. In addition, they observed that the gut microbiota of patients with UTIs was rich in *E. coli* up to 7–14 days post-antibiotic treatment. UPEC isolates from the gut showed an elevated phenotypic resistance against a number of drugs tested, ranging from 11 to 23, compared to non-colonized individuals [10]. Recent studies have shown that an imbalance in the gut microbiota, or dysbiosis, can lead to the production of harmful metabolites, such as lipopolysaccharides (LPS), which further exacerbate gut permeability [25]. Therefore, the gut microbiota can act as a “facilitator”, meaning a dysbiotic gut facilitates UPEC colonization in recurrent UTIs, or as an “agitator”, meaning a dysbiotic gut promotes the activation of the host immune system, resulting in increased inflammation in response to UPEC invasion of the urinary tract, worsening symptoms and severity of UTIs [3,26]. The gut microbiota is also considered a source of uropathogenic *Enterobacterales* and *Enterococcus* that can colonize both the periurethral space and ascend to the bladder, being responsible for dysregulation of host local physiology and inflammation of regional mucous membranes, which are critical for the homeostasis of the urinary tract. These translocated bacteria and their byproducts can trigger a systemic immune response, increasing the susceptibility to infections in other organs, including the bladder [27,28]. There is growing evidence that the bacteria most commonly found in UTIs, such as *Escherichia coli*, may originate from the gut, suggesting a route of translocation from the intestines to the bladder [29]. One proposed mechanism is the direct migration of bacteria through the bloodstream after crossing the compromised gut barrier. Once in circulation, these bacteria can reach and colonize the bladder, particularly in individuals with weakened immune defenses. Additionally, the local immune response in the bladder can be impaired by the systemic inflammation caused by bacterial translocation, making the urinary tract more susceptible to infection. The bladder microbiota, which normally plays a role in preventing infections, can also be disrupted by these translocated bacteria, further increasing the risk of UTIs [30,31]. Understanding the link between leaky gut and bacterial translocation to the bladder has significant clinical implications. Therapeutic strategies that aim to restore gut barrier function and manage dysbiosis may reduce the risk of UTIs. For instance, the use of probiotics and dietary interventions can promote a healthy gut microbiota and enhance the integrity of the gut barrier [32,33,34,35,36,37,38]. These approaches could be particularly beneficial for individuals with recurrent UTIs or underlying gastrointestinal conditions, such as irritable bowel syndrome (IBS) or inflammatory bowel disease (IBD) [39]. Moreover, ongoing research into the role of gut-derived metabolites in modulating immune responses in the bladder could lead to new preventative strategies against UTIs.

Interventions that target the gut microbiota or its metabolites may offer novel therapeutic avenues for reducing bacterial translocation and improving bladder health. Leaky gut syndrome is increasingly recognized as a key factor in the development of systemic diseases, including UTIs. The translocation of bacteria from the gut to the bladder highlights the importance of maintaining intestinal barrier integrity. Future research should focus on elucidating the specific pathways involved in bacterial translocation and developing targeted therapies to prevent this process. Integrating these insights into clinical practice could lead to more effective treatments for patients with recurrent UTIs and other related conditions.

### 1.2. Gut–Bladder Axis in Recurrent UTIs

The pathogenesis of UTIs typically initiates with the contamination of the periurethral area by uropathogens from the gastrointestinal tract, followed by the colonization of the urethra and subsequent ascension to the bladder [1,40]. Studies on urinary microbiomes revealed that *Lactobacillus* and *Streptococcus* are the most frequently observed species with protective roles against pathogens colonizing the urogenital tract. Other less frequently identified bacterial strains are *Saccharofermentans*, *Alloscardovia*, *Veillonella*, *Burkholderia*, and *Jonquetella* [1,41]. Techniques such as 16S rRNA sequencing and advanced quantitative culture of urine have detected the dominance of *Lactobacillus* in the normal vaginal flora. *Lactobacilli* can prevent both the adherence, growth, and colonization of uropathogenic bacteria and have a strong inhibitory effect on UPEC [42]. Some antibiotic treatments (most prescribed are ciprofloxacin, amoxicillin, ceftriaxone, fosfomycin, levofloxacin, trimethoprim/sulfamethoxazole, etc.) can render the natural barrier of the urinary tract vulnerable to infections, and they can produce the shift of Lactobacillus to coliform uropathogens. UPEC accounts for over 80% of community-acquired infections, whereas healthcare-related infections are predominantly caused by *Staphylococcus*, *Klebsiella*, *Enterobacter*, *Proteus*, and *Enterococcus* species (Table 2) [43]. UPEC strains, found in abundance in the intestine of UTI patients, possess extragenetic material encoding genes for pathogenicity factors such as adhesins, toxins, surface polysaccharides, flagella, and iron-acquisition systems [44].

**Figure 1:**
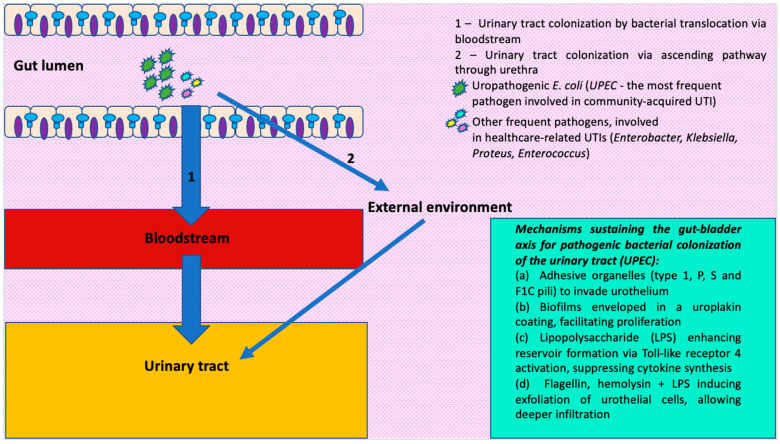
Schematic representation of the mechanisms involved in urinary tract colonization, especially known for uropathogenic *E. coli* (*UPEC*). Figure generated with Microsoft Power Point (Version 16.88.1—License Microsoft 365, © 2024 Microsoft).

UPEC employs adhesive organelles, including type 1, P, S, and F1C pili, to invade host cells within the urothelium (Figure 1). Subsequently, UPEC forms intracellular bacterial communities (IBCs), characterized by biofilms enveloped in a uroplakin coating, which facilitates secure proliferation. A critical element of UPEC pathogenicity, lipopolysaccharide (LPS), influences UPEC life cycles and enhances reservoir formation by triggering intracellular signaling pathways and innate and adaptive immune responses. LPS elevates cytosolic calcium via Toll-like receptor 4 (TLR4) activation, suppressing cytokine synthesis. Additionally, activation of the NLRP3 inflammasome by pathogen-associated molecules such as flagellin, hemolysin, and LPS can induce exfoliation of urothelial cells, allowing deeper UPEC infiltration [45].

Comparative genomic analyses have revealed that UPEC recurrence cycles are driven by urinary persistence, reinfection from external sources, and gastrointestinal colonization, with the gut acting as a known reservoir for UPEC, seeding multiple UTI episodes [46].

UTIs are more common in women due to the anatomical proximity and shorter distance of the female urethra from the anus, facilitating the migration and colonization of gut microorganisms in the urinary tract. A recent study that examined the gut microbiomes of 15 women with a history of recurrent urinary tract infections (rUTIs) and 16 healthy individuals found a decrease in microbial diversity of women with rUTIs, particularly a reduction in butyrate-producing bacteria [47]. Thänert et al. [48] combined semiquantitative culturing with comparative genomics to demonstrate repeated transmission of uropathogens between the gut and urinary tract, indicating that rUTIs are frequently preceded by an “intestinal bloom of uropathogens.” Additionally, previous repeated use of antimicrobials may increase the likelihood of UPEC colonization. Immunological assessments have shown that intracellular bacterial communities (IBCs) and quiescent intracellular reservoirs (QIRs) enable pathogens to survive antibiotic treatment and immune responses in the bladder, leading to chronic colonization [49].

Breastfeeding has been demonstrated to offer protective benefits against urinary tract infections (UTIs) in infants and preterm neonates, which supports the theory that the gut may be a source of UTI origin [50]. A longitudinal case–control study by Hong et al. [51] on preterm infants revealed distinct microbial development trajectories in UTI infants with different pathogens. Notably, there was a significant increase in UTI pathogen-related taxa in the gut before UTI onset, indicating the role of gut flora in UTI susceptibility and its potential as a diagnostic and therapeutic target [52,53].

In a prospective case–control study by Paalanne et al. [54] on the intestinal microbiome as a risk factor for UTIs in the pediatric population, sequencing of the bacterial 16S rRNA gene was performed. The study compared the intestinal microbiomes of 37 children with febrile UTIs and 69 healthy children. While no significant differences were noted at the phylum level, several differences were observed at the family and genus levels, including an enrichment of the genus *Enterobacter* in UTI-affected children’s gut microbiome and of the *Peptostreptococcaceae* family in healthy controls. Additionally, they measured fecal lactoferrin, an antimicrobial transferrin protein also contained in breast milk, hypothesizing its deficiency in the gut environment as a risk factor for UTIs in children [49,50]. However, no association between fecal lactoferrin concentrations and UTI was confirmed in their study [54].

A longitudinal, multicenter cohort study by Choi et al. [10] analyzed stool samples from patients with UTIs caused by antibiotic-resistant organisms [55,56]. Gut microbiome profiles associated with UTIs were compared to previously published microbiomes from healthy individuals and those with UTIs, focusing on taxonomic composition, antimicrobial resistance genes (ARGs), and phenotypic resistance [57]. The UTI group’s gut microbiome was distinct from healthy reference microbiomes in terms of taxonomic composition and ARG burden, showing 11 differentially abundant taxa at the genus level. Although there were no significant differences observed between the gut microbiomes of patients with recurrent UTIs (rUTIs) and those without, the microbiomes of individuals colonized with urinary tract pathogens showed higher levels of *E. coli* abundance 7–14 days after antimicrobial treatment. Gut isolates of UPEC from lineages colonizing the urinary tract displayed higher levels of resistance to 11 out of 23 tested drugs compared to non-colonizing lineages.

The study utilized metagenomics to explore the gut–bladder axis, showing that the gut microbiome of individuals with UTIs differed from that of healthy individuals, reinforcing the link between gut microbiome dysbiosis and UTI development [58,59]. The genera *Akkermansia*, *Bilophila,* and *Parasutterella* were found to be depleted in the intestinal samples of UTI subjects, aligning with earlier studies. However, no significant differences were observed in the gut microbiome between patients who experienced recurrence during the study period and those who did not. Patients with urinary tract colonization had increased gut *E. coli* levels at asymptomatic post-antimicrobial stages. Urinary tract colonization was associated with greater phenotypic resistance in gut isolates, though urinary isolates did not show this pattern. Additionally, Choi’s study [10] linked these observations to the presence of “hidden” ARGs in UPEC lineages, likely acquired through mobile genetic elements enriched in the gut microbiome. While urinary isolates from the same pathogen lineage did not retain high resistance during asymptomatic colonization, highly resistant gut isolates may potentially migrate and trigger recurrent infections in the urinary tract [60].

One suggested approach to addressing recurrent UTIs (rUTIs) is the preventative use of cranberry products, though their effectiveness in preventing UTIs remains uncertain [61]. No specific in vivo mechanism has been identified for cranberry’s potential protective effects, with possible mechanisms only demonstrated in vitro [62,63,64,65]. To investigate the impact of daily cranberry consumption on the gut microbiota, Straub et al. [66] studied the gut microbiome of women with a recent history of rUTIs who consumed either cranberry or placebo beverages daily for 24 weeks. The researchers analyzed 16S rRNA gene and whole metagenome sequencing data from stool samples of 70 women in a randomized, double-blind, placebo-controlled, multicenter clinical trial [67]. The study found that long-term daily cranberry consumption did not lead to significant taxonomic or functional changes in the gut microbiome, but it was associated with a reduction in *Flavonifractor OTU41* compared to long-term placebo consumption. *OTU41* carries functions related to the transport and metabolism of compounds such as tryptophan and cobalamin, important molecules in biochemical pathways that may be linked to UPEC pathogenesis and/or rUTI disease. This research adds to the growing evidence that links increased *Flavonifractor* abundance in the gut to negative health outcomes [68,69,70,71]. As rUTIs become increasingly challenging to treat due to rising antimicrobial resistance, further exploration of cranberry products and their effects on the gut microbiome, including their influence on *OTU41* and resident *E. coli*, is crucial to understanding their impact on rUTI development and outcomes.

Other significant data on the gut–bladder axis are provided by a recent study conducted by Magruder et al. [3], focusing on patients particularly susceptible to bacteriuria and UTI, specifically kidney transplant recipients. In particular, this pilot study aimed to investigate the relationship between gut microbiota and the risk of developing bacteriuria and UTI through serial gut microbiota profiling of fecal specimens from approximately 170 kidney transplant recipients. The authors reported that higher gut abundances of *Escherichia* and *Enterococcus* were associated with the future development of *Escherichia* and *Enterococcus* bacteriuria, respectively, independent of clinical factors such as gender. Furthermore, the gut abundance of *Escherichia* was linked to the development of symptomatic *Escherichia* UTI, whereas no similar relationship was found between *Enterococcus* abundance and *Enterococcus* UTI.

A subsequent study by the same group [72], involving the same number of kidney transplant recipients and using similar methods, assessed the relationship between the relative abundance of commensal taxa of bacteria and the development of bacteriuria and UTI related to *Enterobacteriaceae*. This study found that higher relative abundances of *Faecalibacterium* and *Romboutsia* were linked to a reduced risk of *Enterobacteriaceae* bacteriuria and UTI in kidney transplant recipients. These results support the growing understanding that gut commensal organisms play a role in lowering the risk of infectious complications, a concept already well established for *Clostridioides difficile* infections. The authors suggested that one possible mechanism by which low levels of Faecalibacterium or Romboutsia might prevent the growth of *Enterobacteriaceae* is through the production of short-chain fatty acids (SCFAs). *Faecalibacterium* is a key producer of the SCFA butyrate in the gut, while species within the *Romboutsia* group are also believed to be associated with the production of the SCFA acetate [72,73].

Butyrate, an SCFA produced by bacteria belonging to the gut microbiome, activates the anti-inflammatory signaling pathway of PPAR-γ in the gut’s epithelium [74]. If PPAR-γ signaling is lacking, there is an increase in oxygen levels and nitrate concentration within the lumen because of nitric oxide synthase’s induction and reduced activity of the β-oxidation pathway within epithelial cells. Antibiotic treatment can deplete butyrate-producing taxa, disrupting PPAR-γ signaling and providing a growth advantage to *E. coli* [11,75,76].

### 1.3. Fecal Microbiota Transplantation: A Growing Therapeutic Niche

The aforementioned study by Magruder et al. [72] could not only contribute to the development of personalized probiotic consortia for UTI prevention but also aid in better personalizing fecal microbiota transplantation (FMT) for patients with recurrent *Enterobacteriaceae* UTIs.

There is evidence supporting the potential of FMT as a treatment for recurrent *C. difficile* infection, as well as for some other diseases (e.g., pancreatic disorders) [77,78,79,80], but parallelly there is growing literature data supporting its role in recurrent UTIs. For instance, case reports [81,82] detailed a heart and kidney transplant recipient who had several episodes of *Enterococcus* bacteremia, UTIs, and recurrent *C. difficile* infections. Following FMT, there was a reduction in the fecal abundance of *Enterococcus*, leading to a resolution of the *Enterococcus* infections for up to 14 months. Moreover, a case series [83] involving FMT for recurrent *C. difficile* infections in a non-transplant population demonstrated a notable decrease in the number of UTIs in the year after FMT compared to the previous year.

These findings underscore the importance of gut microbiota in the pathogenesis and potential treatment of UTIs, highlighting the need for further research into microbiome-based therapies.

### 1.4. Probiotics and UTIs

It is known that recurrent UTIs are a common issue, especially among women, and recent studies have highlighted a significant link between these infections and the gut microbiome. Imbalances in the gut microbiota can influence the recurrence of UTIs. Women with recurrent UTIs often have less diverse gut microbiomes and higher levels of inflammation. This imbalance in gut bacteria can make them more susceptible to infections. A recent study on women with a history of UTIs revealed that over 70% of the participants had intestinal dysbiosis, a gut microbiome imbalance linked to recurrent infections. [84,85]. Research indicates that antibiotics used to treat UTIs can disrupt the gut microbiome, leading to a cycle where the infection recurs. This happens because while antibiotics may clear the infection from the bladder, they often leave behind bacteria in the gut. These bacteria can then migrate back to the urinary tract, causing another infection [47,86]. A recent paper published in *EClinicalMedicine* [10] that compared gut microbiomes in women affected by recurrent UTIs or UTIs and controls highlighted that the species richness was lower among UTI samples compared to healthy controls. Some species such as *Akkermansia* and *Bilophila* are depleted in UTI samples; on the other hand, the healthy samples were enriched in commensal Firmicutes *Roseburia*, *Eubacterium,* and *Ruminococcus*. Additional important evidence is that the gut microbiomes of patients with rUTIs and those without (non-rUTI) are similar [10]. Conventional treatment of UTI usually involves antibiotics, which can be effective in clearing the infection. However, the overuse of antibiotics has led to increasing antibiotic resistance, making it crucial to explore alternative or complementary treatments [7,87].

One promising alternative is the use of probiotics [88,89]. Probiotics, which are live microorganisms, offer health benefits to the host when provided in adequate amounts. These beneficial bacteria are known to play an important role in maintaining gut health, but recent studies suggest that they may also help prevent and treat UTIs. The idea is that probiotics can help restore and maintain the natural balance of bacteria in the urinary tract, inhibiting the growth of harmful bacteria that cause infections. Interestingly, some studies suggest that maintaining a healthy gut microbiome through probiotics and dietary changes might help reduce the risk of recurrent UTIs [90]. This approach aims to restore the balance of beneficial bacteria in the gut, potentially preventing the cycle of infection and antibiotic use. Like the gastrointestinal system, the urogenital tract harbors a wide range of microorganisms that modify pH levels and local metabolite concentrations, which are essential for maintaining the equilibrium of the urine microenvironment [91]. Studies have shown that urinary microorganisms differ between healthy individuals and those with UTIs, with *Lactobacillus* species, which produce lactic acid and lower vaginal pH, often decreasing in patients with UTIs [92]. The most effective probiotics for UTIs are typically strains of *Lactobacillus*, as they are closely linked to urinary tract health. The greatest efficacy has been observed with strains such as *L. rhamnosus GR-1*, *L. reuteri B-54*, *L. reuteri RC-14*, *L. casei shirota*, and *L. crispatus CTV-05*. As a result, the 2022 EAU guidelines recommend probiotics containing these strains for the prevention of recurrent UTIs [93].

In the literature we found several studies that have evaluated the efficacy of different types of probiotics both in preventing the recurrence of UTIs and in treating the acute phase with different results [94,95,96]. One clinical trial included 252 women who had recurrent UTIs. Over a 12-month period, women who took a combination of probiotics found them to be just as effective as antibiotic treatment for cystitis but without the associated side effects [97]. Recent research on urine from healthy women has shown that the bladder microbiome closely resembles the vaginal microbiome, although with a lower biomass [98,99]. The most common genera were *Lactobacillus*, *Gardnerella*, and *Streptococcus*. Within the *Lactobacillus* species, four dominate the bladder microbiome: *L. crispatus*, *L. gasseri*, *L. iners*, and *L. jensenii.* In healthy women, the premenopausal and postmenopausal stages can have different compositions of typical vaginal commensals. In healthy premenopausal women, *Lactobacillus* species typically predominate particularly in women of European heritage as opposed to African American women [100].

These modifications are caused by several reasons, including shifts in estrogen levels, vaginal pH, glycogen levels, and the menstrual cycle [101]. These elements work together to influence how infections colonize and adhere to vaginal epithelial cells. In healthy premenopausal women, elevated estrogen levels promote *Lactobacilli* adhesion while inhibiting the colonization of other pathogens. On the other hand, as estrogen levels drop in postmenopausal women [102], the vaginal microbiota changes and may become more susceptible to urogenital infections. According to Barrea, probiotics are a viable strategy for reestablishing the balance of commensal organisms in the vagina, which can reduce the creation of biofilms and hinder the proliferation of pathogenic organisms [103]. Since lactobacillus species are designed to restore the vaginal microbiota, they should be included in probiotics that prevent and treat genitourinary infections. Probiotics may work by creating nutrients, including vitamins and immunomodulators, acidifying the mucosal surface, inhibiting pathogen adherence, and working in a complimentary manner with the host’s immune system. Certain *Lactobacillus* species produce hydrogen peroxide and biosurfactants, which cause the vaginal mucosa to become acidic. These traits have been demonstrated to have regulatory ramifications that are micro biomimetic. For all these reasons, lactobacillus is the probiotic that is advised for the treatment and prevention of urogynecologic diseases [103]. Reid G et al. [104] conducted significant research in 2001 on the use of *Lactobacillus rhamnosus*, specifically strain GR-1, for the prevention and treatment of UTIs. His studies have shown that this probiotic strain can effectively reduce the recurrence of UTIs. One of the key findings is that vaginal suppositories containing *L. rhamnosus* GR-1 and *L. fermentum* B-54 can reduce the recurrence rate of UTIs in women who have treated a first episode with antibiotics. Additionally, the oral administration of *L. rhamnosus* GR-1, in combination with other *Lactobacillus* strains, has shown improvements in vaginal flora, reducing the presence of harmful bacteria and increasing the prevalence of beneficial lactobacilli [105]. By producing antimicrobial agents like bacteriocins and biosurfactants that change the surface tension of the surrounding fluid, *Lactobacillus* sp. inhibit vaginal pathogens by preventing adhesion and further limiting their spread in the bladder. Additionally, lactobacilli are essential for preserving the pH of the vagina [106,107]. Studies have demonstrated that giving premenopausal women the probiotic *Lactobacillus rhamnosus* GR-1 can raise the expression levels of antimicrobial defenses. In a double-blind, randomized, and placebo-controlled study, 81 adult premenopausal women with recurrent UTIs participated. The purpose of the study [108] was to assess a product that contained two *Lactobacilli* and cranberry extract to prevent recurrent UTIs in premenopausal women. At the end of treatment, they obtained a prevention in recurrent UTIs in the supplemented group without side effects. Stapleton et al. [109] in 2011 published in the “Clinical Infectious Diseases” journal a study in which investigated 100 premenopausal women who had undergone cystitis at least once within the last 12 months. They evaluated the efficacy of the intravaginal administration of *Lactobacillus crispatus* CTV-05 (LACTIN-V) (108 CFUs/mL) compared to placebo. The study found that women who received the probiotic had a significantly reduced recurrence of UTIs compared to the placebo group. The study highlighted the potential of *L. crispatus* in re-establishing a healthy vaginal microbiota and reducing UTI risk [109].

A complex of five probiotic strains were tested in another clinical trial to evaluate if they could help 76 women who were of reproductive age in restoring their vaginal health [110,111]. The ensuing investigations verified the increase in *L. plantarum* together with the inhibition of pathogenic bacteria such as *Mobiluncus* species, *Gardnerella vaginalis*, and *Atopobium vaginae*. Because it enhances the vaginal microbiota, this probiotic combination has been shown in a clinical trial to be useful in the treatment of bacterial vaginosis [112]. Most of the research in the literature on the use of probiotics for recurrent UTIs has been performed on premenopausal women. Premenopausal women have different risk factors than postmenopausal women. A history of UTIs, the use of spiral or condoms containing spermicide, and recent antibiotic use are some of these risk factors. Additional risk factors include those that lead to the colonization of uropathogens in the vagina and a reduction in the number of lactobacilli that enter the urine system through the ascending pathway.

Single or combination treatments with probiotics have been applied with different strains and different routes of administration (vaginal, oral), and effective results have been reported [113,114]. Montorsi et al., in a prospective study, administered a combination of vitamin C, *L. rhamnosus,* and cranberry three times daily for 10 days and then repeated the whole cycle three times. After 3 and 6 months, around 70% remained asymptomatic with a negative urine culture [115]. The largest randomized controlled trial on oral probiotics for recurrent UTIs was conducted by Beerepoot et al. [97] in 2012. This study compared 12 months of daily trimethoprim/sulfamethoxazole (TMP-SMX) to twice-daily oral doses of *L. rhamnosus GR-1* and *L. reuteri RC-14* in 252 women with recurrent UTIs. Both groups experienced a decrease in the total number of UTIs, dropping from 7.0 to 2.9 UTIs per year in the antibiotic group and from 6.8 to 3.3 UTIs per year in the probiotic group. However, the probiotic treatment did not achieve the 10% difference between groups required to meet the predefined noninferiority criteria. As anticipated, resistance in *E. coli* to TMP-SMX, TMP, and amoxicillin increased by approximately 55–60% within a month of treatment. The authors highlighted this as an additional factor to consider when calculating the benefit-to-risk ratio of the two interventions. Over a 12-month period, no changes in the vaginal microbiome were observed in either the probiotic or antibiotic groups. *L. reuteri* was not detected in the vagina at either time-point [97]. A recent double-blind, placebo-controlled study [116] was performed on premenopausal women with a history of recurrent UTIs. Patients were randomized into four different treatments with oral or vaginal lactic acid bacteria and bifidobacteria compared to oral or vaginal placebo for 4 months. Prophylactic supplementation, either with vaginal probiotics alone or in combination with oral probiotics, proved effective in preventing recurrent symptomatic UTI episodes during a one-year follow-up. A comprehensive Cochrane review [89], which included nine studies with a total of 735 participants, found no significant reduction in the risk of recurrent symptomatic bacterial UTIs between patients treated with probiotics and those given a placebo. The relative risk (RR) was 0.82, with a 95% confidence interval (CI) of 0.60 to 1.12, indicating no substantial difference. Additionally, when compared with antibiotic treatment, probiotics did not show a significant benefit (RR 1.12, 95% CI 0.95 to 1.33). Another systematic review and meta-analysis [117] published in *BMC Infectious Diseases* also highlighted the potential role of probiotics as adjuvants in treating infections, including UTIs. This review noted some studies showing benefits of probiotics in reducing UTI recurrence, but the overall evidence was not strong enough to draw definitive conclusions due to the variability in study designs and probiotic strains used. Moreover, other reviews explored the use of *Lactobacillus* species and other probiotics in preventing recurrent UTIs, suggesting potential benefits [118,119,120]. However, the studies reviewed often had limitations such as small sample sizes and methodological biases and different kinds of probiotics with different dosages and for different periods of consumption, which complicates the interpretation of results. A limit of our study is that, despite the quantity of studies analyzed and a rigorous application of inclusion and exclusion criteria, the still scarce number of RCTs on the use of probiotics for rUTIs did not allow us to conduct a statistical analysis aimed at generating a systematic review with an at least moderate statistical power.

**Table 1:** **Studies about the relationship between gut microbiota and rUTIs.**

Magruder et al. (2019) [3]	Authors performed gut microbial analysis using 16S rRNA gene sequencing on 510 fecal sample from 168 kidney transplant recipients and metagenomic sequencing on a subset of fecal specimens and urine supernatant specimens.	Results supported the association between gut microbiota–UTI, suggesting that modulating the gut microbiota may be a strategy to prevent UTIs.
Choi et al. (2024) [10]	125 patients with UTI enrolled from July 2016 to May 2019; 644 stool samples and 895 UPEC isolates.	The gut microbiome is implicated in the colonization of the urinary tract by UPEC during rUTI. It serves as a reservoir for UPEC.
Hashemizadeh et al. (2017) [24]	Authors analyzed the various virulence factors of UPEC with molecular analysis of fecal samples.	The majority of examined virulence genes which are important in establishment and maintenance of infections were found to be more common in fecal samples of patients with UTIs.
Worby at al. (2022) [47]	A year-long study of women with (n = 15) and without (n = 16) history of rUTI, from whom authors collected urine, blood, and monthly fecal samples for metagenomic and transcriptomic analysis.	Results suggest that rUTI susceptibility is in part mediated through the gut–bladder axis, comprising gut dysbiosis and differential immune response to bacterial bladder colonization.
Thanert et al. (2019) [48]	Fourteen patients (median: 63 years; from 37 to 88 years) with symptomatic UTIs caused by antibiotics resistent (AR) uropathogens were enrolled in this study	There is evidence of repeated transmission of uropathogens between the intestinal reservoir and the urinary tract and evidence that rUTIs are frequently preceded by an intestinal bloom of uropathogens.
Paalanne et al. (2018) [54]	Prospective case–control study compared the gut microbiomes of 37 children with a febrile UTI with 69 healthy children.	The risk of UTI and pyelonephritis in children could be associated with the intestinal environment and its gut microbiome.
Ruta et al. (2024) [84]	Fecal dysbiosis tests were performed comparatively in two groups of women.	Gut dysbiosis can have an impact on the recurrence of urinary tract infections.

**Table 2:** **Main type of uropathogens: characteristics and mode of action.**

Pathogens	Mechanism of Action	Prevalence (%)	Gram Stain	Mode of Infection
*UPEC*	FimH adhesin Type 1 fimbriae Type 2, P fimbriae Dr family adhesins S fimbriae F1C	55–70%	negative	Community acquired
*Enterococci*	Ebp pili GelE and SprE proteases Enterococcal Surface Protein	5–7%	negative	Nosocomial
*Staphylococci*	Factor B (ClfB)	2–5%	positive	Community acquired
*Proteus mirabilis*	(MR/P) fimbriae NAF fimbriae Mrp/H	5–10%	negative	Catheterized patients
*Klebsiella pneumoniae*	Type 1 fimbriae Type 3 fimbriae	25–35%	negative	Healthcare associated opportunistic
*Pseudomonas aeruginosa*	T4Pa	8–15%	positive	Nosocomial

UPEC, uropathogenic *Escherichia coli*; F1C, type 1-like immunological group C pili; T4Pa, type IV pili; MRP, mannose-resistant Proteus fimbriae; NAF, non-agglutinating fimbriae; Aas, autolysin/adhesin of *Staphylococcus saprophyticus*; SdrI, *serine*–aspartate repeat proteins; Uaf, uro-adherence factor.

## 2. Conclusions

Recent microbiological discoveries are providing an entirely new pathophysiological picture of recurrent UTIs compared to ten years ago. Nowadays, it is clear how in the pathogenesis of UTIs the microbiome plays a central role and how, consequently, the exclusive use of antibiotics is not only ineffective but also counterproductive. Antibiotics may indeed eradicate the bacterial infection, but they create intestinal dysbiosis, triggering a vicious circle. Interventions that target the gut microbiota or its metabolites may offer novel therapeutic avenues for reducing bacterial translocation and improving bladder health. Future research should focus on elucidating the specific pathways involved in bacterial translocation and developing targeted therapies to prevent this process. Integrating these insights into clinical practice could lead to more effective treatments for patients with recurrent UTIs and other related conditions.
